# Multifactor consciousness level assessment of participants with acquired brain injuries employing human–computer interfaces

**DOI:** 10.1186/s12938-019-0746-y

**Published:** 2020-01-10

**Authors:** Andrzej Czyżewski, Adam Kurowski, Piotr Odya, Piotr Szczuko

**Affiliations:** Multimedia Systems Department, Faculty of Electronics, Telecommunications and Informatics, Gdańsk University of Technology, Gdańsk, Poland

**Keywords:** Acquired brainstem response, Multimedia computers, Human–computer interfaces, Eye tracking, Electroencephalography, Auditory brainstem injuries, Data clustering analysis

## Abstract

**Background:**

A lack of communication with people suffering from acquired brain injuries may lead to drawing erroneous conclusions regarding the diagnosis or therapy of patients. Information technology and neuroscience make it possible to enhance the diagnostic and rehabilitation process of patients with traumatic brain injury or post-hypoxia. In this paper, we present a new method for evaluation possibility of communication and the assessment of such patients’ state employing future generation computers extended with advanced human–machine interfaces.

**Methods:**

First, the hearing abilities of 33 participants in the state of coma were evaluated using auditory brainstem response measurements (ABR). Next, a series of interactive computer-based exercise sessions were performed with the therapist’s assistance. Participants’ actions were monitored with an eye-gaze tracking (EGT) device and with an electroencephalogram EEG monitoring headset. The data gathered were processed with the use of data clustering techniques.

**Results:**

Analysis showed that the data gathered and the computer-based methods developed for their processing are suitable for evaluating the participants’ responses to stimuli. Parameters obtained from EEG signals and eye-tracker data were correlated with Glasgow Coma Scale (GCS) scores and enabled separation between GCS-related classes. The results show that in the EEG and eye-tracker signals, there are specific consciousness-related states discoverable. We observe them as outliers in diagrams on the decision space generated by the autoencoder. For this reason, the numerical variable that separates particular groups of people with the same GCS is the variance of the distance of points from the cluster center that the autoencoder generates. The higher the GCS score, the greater the variance in most cases. The results proved to be statistically significant in this context.

**Conclusions:**

The results indicate that the method proposed may help to assess the consciousness state of participants in an objective manner.

## Background

The incidence rate of traumatic brain injuries (TBI) in Europe in 2006 was reported as 235 cases per 100,000 people per year [1]. An efficient tool for communicating with such patients and for evaluating their communication abilities is needed. Unfortunately, both communication and assessment of the progress made in the course of care by participants with disorders such as TBI, sudden cardiac arrest (SCA), and hypoxia may pose a real challenge to a therapist. All the groups of disorders mentioned could collectively be called acquired brain injuries (ABI). Typical procedures concerning participants with TBI and other disorders include examination employing one of the various imaging techniques. The current literature indicates several common ways to assess consciousness in people suffering from acquired brain injuries ABI. Many of them involve some use of EEG signal measurement, however, techniques such as transcranial stimulation, deep brain stimulation are also possible, and they were described in the literature [2]. EEG-based techniques often apply measurement and analysis of event-related potentials (ERP) [2]. They also may involve using external stimuli such as auditory signals [3]. Another example of an EEG-based technique is a mismatch negativity (MMN) approach [4, 5]. Over the last two decades, computed tomography (CT) and magnetic resonance imaging (MRI) scans have become standard medical practice, and they are widely used for many aspects of modern medicine, like for example detection of tumors or identification of sites of injury from impact [6–8]. Although positron emission tomography (PET) and functional magnetic resonance imaging fMRI are also employed for medical diagnostics, they are not standard medical procedures, because of their high cost, thus they are more often used for research purposes [9]. Some works also identify the need for cheap and wide-applicable solutions for the estimation of the state of patients suffering from ABI [2]. Researchers and physicians have proved that the use of PET and especially fMRI is eventually inevitable in the clinical examination of patients with disorders of consciousness [10–13] due to their accuracy and usability. However, high cost is still the factor that significantly limits their use for daily care, especially outside hospitals. Thus, a less expensive and more portable solution is required, which would be capable of evaluating the response of participants to stimulation performed reliably by a therapist and of improving the understanding of their current needs.

Some studies have concluded that in case of communication with patients with locked-in syndrome, it may be possible to employ EEG [14] or eye-tracker data [15] as a means of interaction. Some research also suggests that long-term monitoring of EEG signals gathered when participants are sleeping may help to predict the outcome of the therapeutic process [16]. Recently, an approach applying an eye-tracker device was employed to assess the emotional states of healthy participants [17]. There are also works that identify the need for cheap and widely applicable solutions for the estimation of the state of patients suffering from ABI [2]. We would like to propose a direction of diagnosis based upon the bimodal measurement of the activity of people taking part in therapeutic sessions. Participants of our research suffered from acquired brain injuries, and they took part in therapeutic sessions which involved interaction with computer by using the eye-gaze tracking device (EGT). During those sessions, we measured EEG signals and collected data from the eye tracker. Next, we employed an autoencoder neural network to perform clustering and separability analysis of data collected in the experiment. We were especially interested in employing this method for session-to-session assessment of GCS score, as the literature indicates, that such comparison of GCS values over time may provide valuable clinical information to be used for monitoring the state of patients [18]. This kind of assessment tool may improve therapists’ communication with their patients entailing more effective communication which can help, in turn, to provide reliable and a capacity to objectively evaluate the patients’ state.

The structure of this article is as follows: in the next section results from the analysis of signals obtained from the EEG and eye-tracker devices are presented together with outcomes of the statistical analysis; next, a discussion and conclusions drawn from the results observed are presented in two consecutive sections; the last section provides a brief profile of participants who took part in the experiments together with methodology for verifying the hearing abilities of participants. It also provides a detailed description of the methodology used for making measurements and processing of the data obtained from the experiment participants.

## Results

**Fig. 1:**
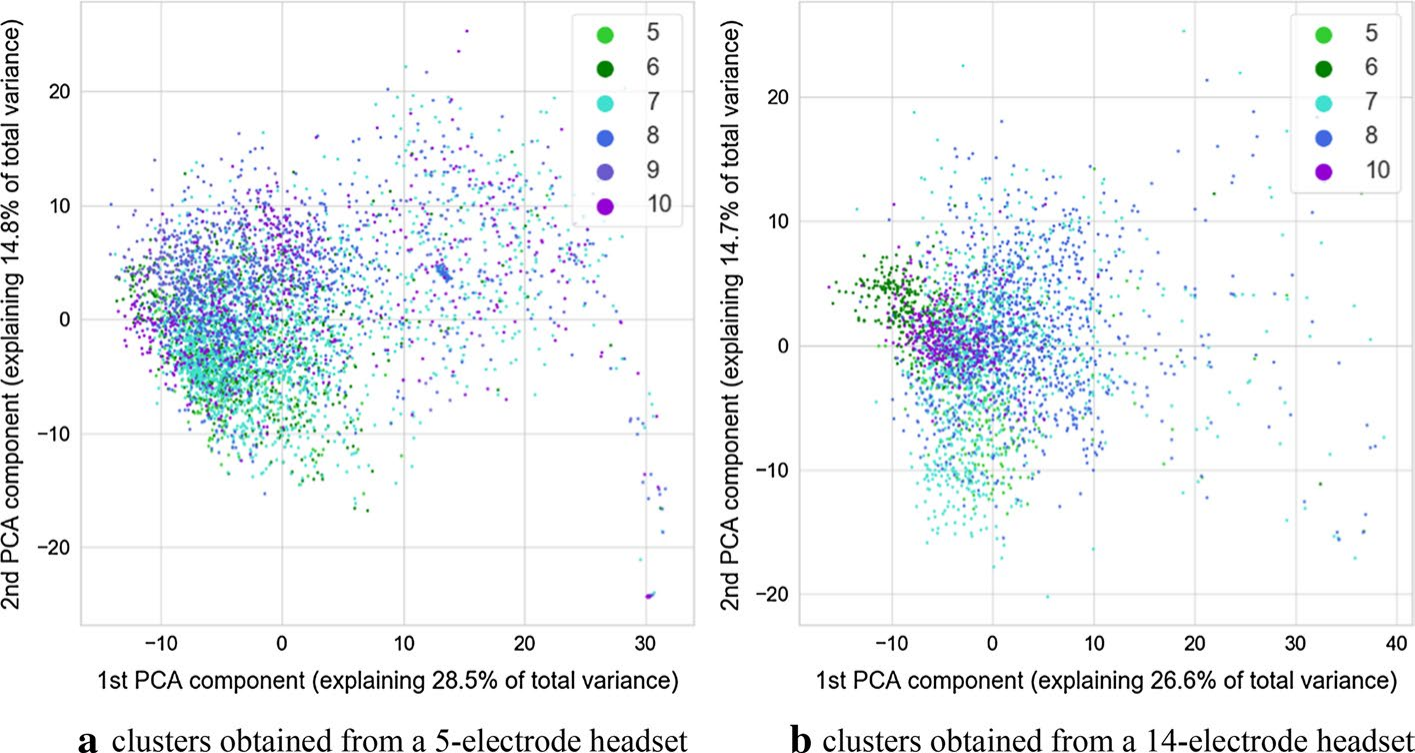
Image of clusters obtained from the autoencoder with different values of GCS score marked with color

**Fig. 2:**
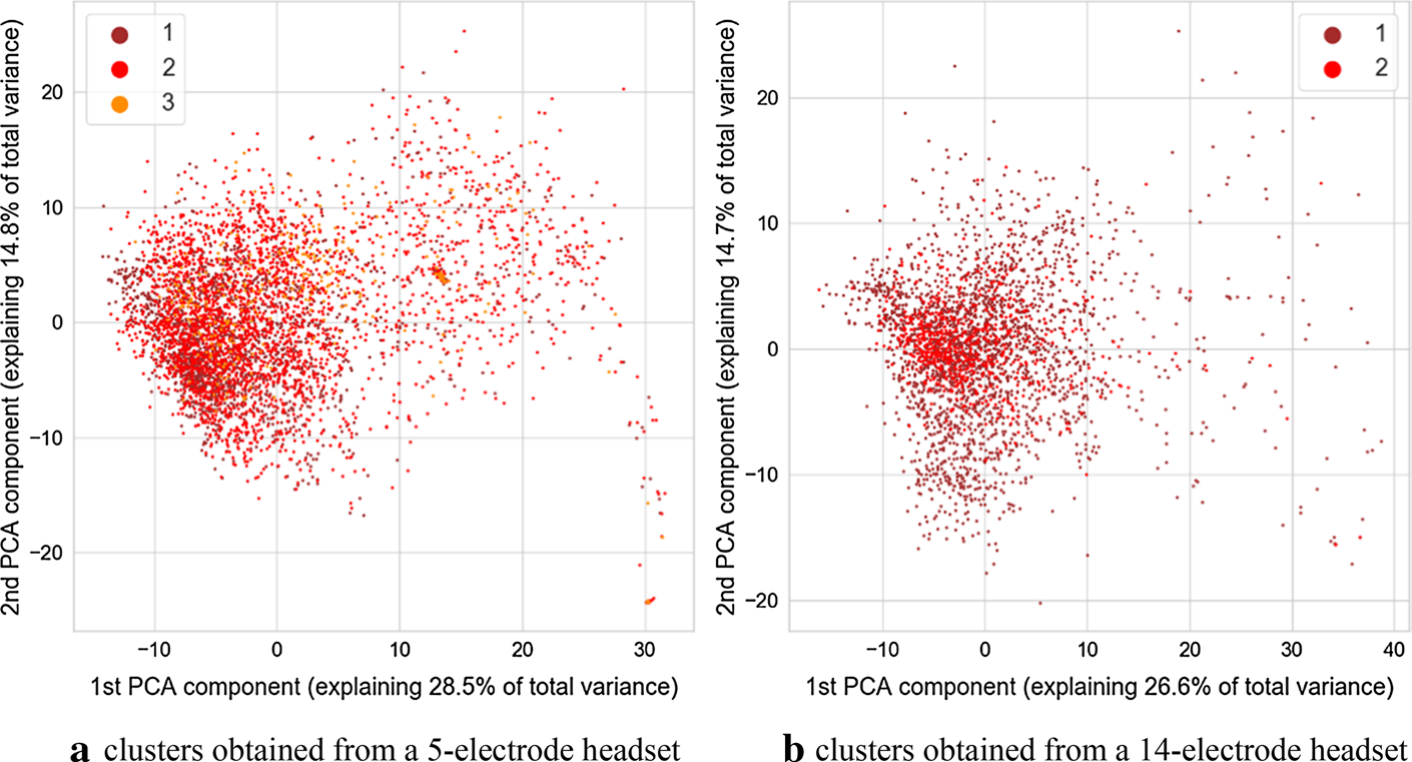
Image of clusters obtained from the autoencoder with different values of the verbal response component of GCS marked with color. Value of 1 indicates making no sounds, 2—the ability to make sounds, and 3—the ability to say single words

**Fig. 3:**
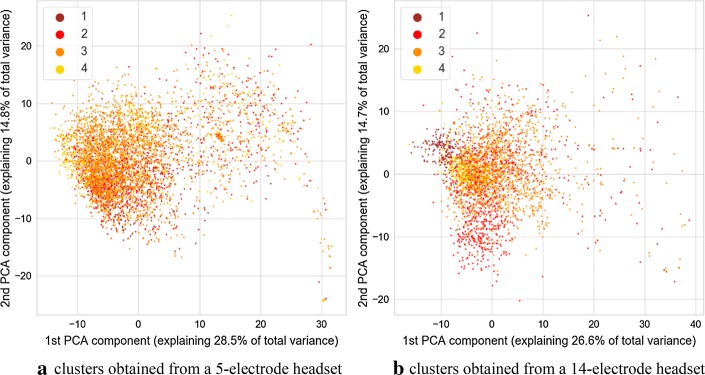
Image of clusters obtained from the autoencoder with different values of the motoric component of GCS marked with color. Value of 1 indicates that participant does not move, 2—extends limb in response to painful stimuli, 3—abnormally flexes a limp in response to painful stimuli, and 4—flexion/withdrawal in response to painful stimuli

**Fig. 4:**
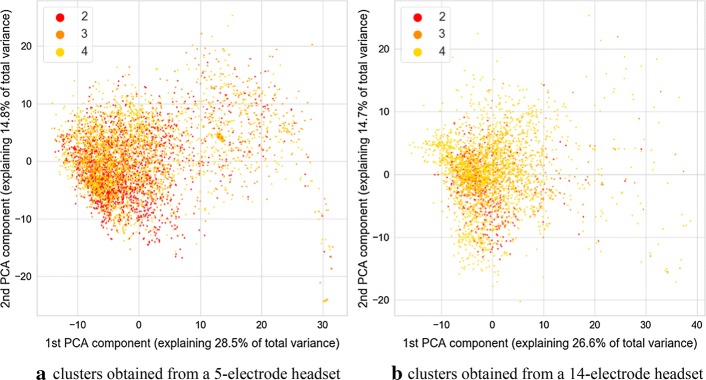
Image of clusters obtained from the autoencoder with different values of eye component of GCS marked with color. Value of 2 indicates that participants can open eyes, 3—opens eyes in response to voice, 4—opens eyes spontaneously

**Fig. 5:**
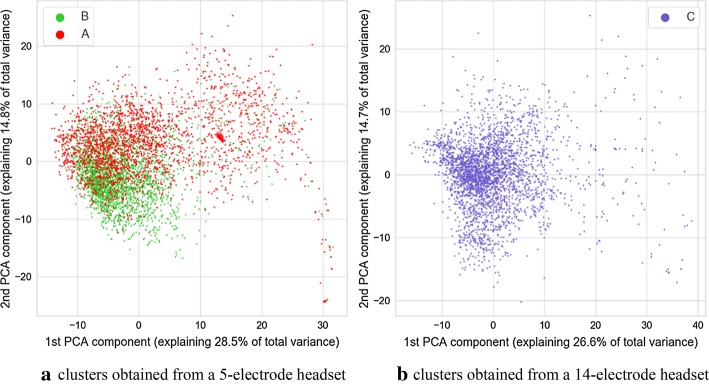
Image of clusters obtained from the autoencoder with three sessions of data acquisition marked with color

The signals gathered during therapeutic sessions performed with the study participants were split into a series of 12-s-long epochs. Each epoch contained signals from both the EEG headset and the eye-tracker device. It was then used to train the multimodal autoencoder neural network, which performed unsupervised data segmentation. Data were collected with two headsets consisting of 5 and 14 electrodes. A separate analysis was performed for each dataset. The only difference between neural networks employed for this purpose is number of EEG-related channels in each network. In each network this number of channels was equal to the number of electrodes in the processed dataset. The encoder part of the autoencoder was utilized to generate vectors of parameters associated with pairs of epochs extracted from EEG and EGT signals. To visualize them on a two-dimensional space, a PCA-based projection onto a plane was determined. Additionally, values of GCS and its components associated with participants from whom the epochs of signals are gathered are marked with color on those figures. Visualizations are shown in Figs. 1, 2, 3, 4, 5.

It is discernible in the coloring of points associated with three sessions of data acquisition marked consecutively as A, B, and C. This visualization is visible in Fig. 5. During the session A, only a 5-electrode headset was used. Both headsets were employed during the session B, and during the session C, only the 14-electrode headset was employed. Such a situation permits an interesting comparison for separability of GCS classes for parameters derived from 5-channel headset and 14-channel headset. Separation of classes is more prominent for the cluster associated with a 14-electrode headset. This is especially prominent for verbal and motoric components of GCS scale. There is also a separation between classes for the cluster related to a dataset gathered with a 5-electrode headset, however it is less pronounced. It should be noted that no information about GCS was delivered to the algorithm, therefore all the separation between GCS-related classes are the result of the training process of the multimodal autoencoder.

In the case of both headsets, the feature which is causing the separation is the variance of clusters associated with each GCS. This can support the hypothesis that for two groups with different GCS values, various amounts of outliers are present, and thus, there is a difference in the variance of data points. The variance of distance between the point and the cluster center was calculated for the 32-dimensional set of vectors produced by neural networks. The calculation was carried out separately for each of headset-related dataset and each unique value of GCS or its component. To test the statistical significance of results, the Brown–Forsythe statistic test was employed. This test checks if variances of multiple groups of observations are different. The calculation was also performed separately for each cluster (associated with 5 or 14 electrodes), and each GCS value within that cluster. As a post hoc test after the Brown–Forsythe test, a modified Dunn test was used. Under standard circumstances, the Dunn test is employed for repeated testing of equality of medians of multiple groups of observations. However, it can also be employed as a post hoc variant of the Brown–Forsythe test, which is equivalent to the ANOVA test performed on vectors of observations transformed in the following way:

1 $$\varvec{x} = \left| {\varvec{x} - \bar{\varvec{x}}} \right|,$$

where $\varvec{x}$ is a vector of observations, and $\bar{\varvec{x}}$ is a mean value of this vector.

As the processing was carried out separately for dataset originating from the 5-electrode headset and 14-electrode headset, statistical analyses were also performed separately for the 32-dimensional result vectors obtained from the encoding part of autoencoder neural networks. Values of variances calculated for each cluster and each value of GCS are depicted in Table 1. Results of calculations related to statistic test are shown in Table 2. It can be observed that many differences seen in values of variance are statistically significant for both the standard significance factor of 0.05 and even 0.001. From Table 1 it also can be observed, that in most cases, the variance of distance between points and center of the cluster increases with the value of GCS. As it can also be observed in the visualization from Figs. 1, 2, 3, 4, this may not be true for all cases. Some of the classes are also separated by the decreasing values of the variance, and points associated with a higher GCS value are concentrated near the center of the cluster.

**Table 1:** Variance of distance between of center of cluster (associated with 5 or 14-electrode headset) and points associated with given GCS values, $n_{\text{ex}}$ denotes number of points used for variance calculation

5 electrodes, GCS (sum)			5 electrodes, GCS (eyes)			5 electrodes, GCS (motor)			5 electrodes, GCS (verbal)		
GCS	Variance	$n_{\text{ex}}$	GCS	Variance	$n_{\text{ex}}$	GCS	Variance	$n_{\text{ex}}$	GCS	Variance	$n_{\text{ex}}$
5	12.860	410	2	29.211	1835	1	18.581	336	1	37.107	1915
6	21.247	592	3	71.507	1295	2	67.480	1259	2	58.221	2758
7	55.822	1953	4	55.497	1876	3	42.782	2777	3	65.401	333
8	61.848	1046				4	72.903	634			
9	37.872	841									
10	79.022	524									

14 electrodes, GCS (sum)			14 electrodes, GCS (eyes)			14 electrodes, GCS (motor)			14 electrodes, GCS (verbal)		
GCS	Variance	$n_{\text{ex}}$	GCS	Variance	$n_{\text{ex}}$	GCS	Variance	$n_{\text{ex}}$	GCS	Variance	$n_{\text{ex}}$
5	32.239	349	2	32.239	349	1	21.061	281	1	52.001	2361
6	21.061	281	3	53.590	588	2	51.859	1088	2	39.748	639
7	57.074	1006	4	52.022	2063	3	60.515	1313			
8	63.194	1046				4	18.385	318			
10	18.385	318									

Calculations were performed for points in a 32-dimensional space of autoencoder-generated embedding vectors

**Table 2:**
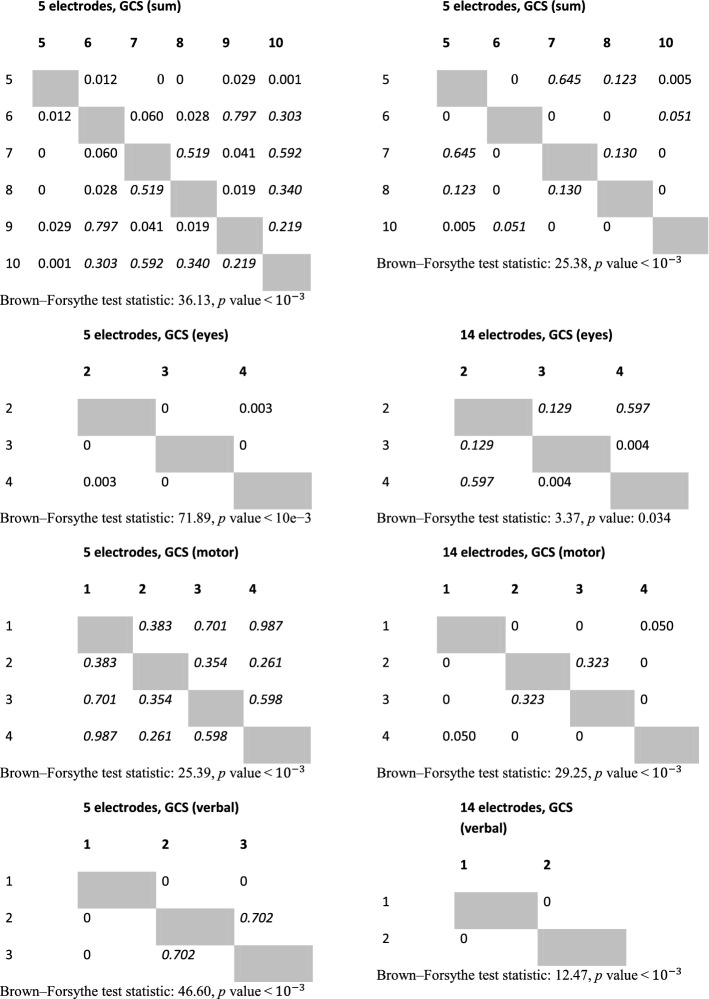
Results of Brown–Forsythe test and *p* value matrices for the following post hoc test

The value of 0 in the table indicates that the *p* value was lesser than $10^{ - 3}$. Statistically insignificant values are marked with a italic font

We hypothesize that the variance increases because of the occurrence of unique states of EEG signal, which are mapped by the autoencoder onto a more distant location when compared to more common states. The occurrence of such rare states may be both an indicator of higher or lower GCS value, and their presence can be tracked and treated as a clue about current GCS value associated with each participant. In the case of 5-electrode headset and motoric component of GCS, a statistical significance was implied by Brown–Forsythe test, but none of the pairs compared by Dunn test were found to be statistically different in the Dunn test. This is probably a consequence of unbalanced count of examples, as it can be seen in Table 1. We assume that all the differences, in this case, were statistically insignificant.

## Discussion

The principal purpose of this study was to propose and to verify a set of procedures that could improve the process of assessing the consciousness level of TBI and SCA patients and of those who have experienced brain hypoxia. The first stage of our method was to administer hearing tests: objective hearing tests were preferred over subjective ones because the type of injury prevents normal communication with patients necessary to organize subjective hearing tests. Objective hearing tests were carried out to select participants who might have been able to receive verbal information. These patients participated in mental exercises allowing for an evaluation of their mental state, employing data from the system used for performing tasks formulated by the therapist.

We found that some of the parameters calculated based on signals gathered from the eye tracker and the EEG headset are associated with the GCS scores or with their component values. We identified that in the hyperspace generated by the autoencoder employed for the unsupervised data analysis a variance of distance between points in hyperspace and center of a cluster is associated with the value of GCS and its components. This outcome was confirmed by statistical analysis. The variance of the subset may both increase or decrease with the value of GCS of its components, however, placement of points in the decision space usually allows partial separation between GCS-related classes and this may be utilized as a clue to estimate GCS-related score of patients. Such separation is especially visible in the case of GCS components related to motoric and verbal response. The above changes of variance may be caused by the occurrence of additional, “atypical” vectors of parameters caused by atypical pairs of EEG and eye tracker signal-related frames at the input of an autoencoder. They are different from the most frequent epochs which were placed in proximity of the center of each headsets cluster. Such atypical epochs may indicate rare “states” of EEG and eye-tracker signals which occur only in signals collected from participants with higher GCS values. Thus, the value of variance is increased if their signals are analyzed.

The analysis of mental activities derived from EEG signal may open new ways of assessing engagement and the mental comfort of participants during the mental exercise making and also provide new methods to evaluate their therapy progress through the use of the association of parameters variance identified in our study with some selected components of the GCS score. There are some practical difficulties that have to be overcome before a more robust system for diagnosing, stimulating, and making mental exercises by participants with traumatic brain injuries is developed. The EEG headset has to be less sensitive to small movements from the patients and imperfect skin contact. The dry electrode-based setup of the INSIGHT EEG helmet does not satisfy the above condition, therefore for future research, the use of a saline-based setup is recommended. Despite that fact, we found that tendencies in the variance changes are also observed in signals collected from the 5-electrode headset. The drawback of such heterogeneity in data is the fact that data from the 5-electrode and 14-electrode headsets were assigned to 2 separate clusters.

## Conclusions

We proposed a method for assessing the state of persons with ABI based on the assessment of their hearing abilities and on checking the performance of participants during mental exercises supervised by a therapist employing a multimedia computer extended with HCI devices. The proposed solution was mainly designed for use in daily therapeutic practice. Moreover, the cost-effective choice of components of the proposed system (employing ABR, EGT, and EEG devices) makes it possible to perform diagnostics of patients with brain injury even on a limited budget. As a result, more therapists may obtain the technology to examine the patients’ state objectively and reliably and to establish or maintain communication with them.

A series of tests with a larger group of participants with varying severity of ABI is planned. These tests will permit the investigation of the structure of mental states detected by the algorithm in the context of personal differences associated with the ability to use BCI [19–21]. It would also be worth investigating how the pattern and presence of rarely observed signal detected correlate with the effectiveness of using BCI and if there are participants for whom such a method of monitoring brain activity will not work. This kind of person-specific problem is particularly significant in the field of brain–computer interfaces, where it is called “BCI illiteracy” [22].

Time-domain data associated with changes in the participants’ mental state during the therapeutic session may also deliver vital information for the therapist. We believe that the algorithm present in this study may help in the parameterization of the EEG signal for purposes of classification and evaluation of patients’ state in clinical practice. For instance, such parameterized data can be utilized to identify moments when a participant feels uncomfortable, or he or she is distracted by some external factor. Moreover, the occurrence of repetitive patterns of brain activity correlated with types of exercises performed may be a premise for concluding that a participant is actively, mentally involved in the process of communication. The ability to focus an eye on certain points during the exercise provides a similar kind of premise. The EEG signal analysis method may be used for verification of, or for searching for, some physiological reactions of participants that can be, in turn, associated with the selection of stimuli provided by a therapist. The results are encouraging because, in principle, they show that in the EEG and eye-tracker signals, there are specific consciousness-related states discoverable. We observe them as outliers in diagrams on the decision space generated by the autoencoder. For this reason, the numerical variable that separates particular groups of people with the same GCS is the variance of the distance of points from the cluster center that the autoencoder generates. The higher the GCS score, the greater the variance in most cases. We managed to prove that the results are statistically significant in this context.

Further development of tools for data analysis is also envisaged. Namely, we would like to evaluate the performance of various classifiers for the prediction of participants’ state and for detection of events such as the feeling of discomfort and state of being distracted from the exercise by external factors. Also, more complicated pre-processing of eye-tracker data may be implemented. A recurrent neural network may be used to capture not only the probability distribution of eye gaze placement, but also track the whole trajectory of the eye-fixation point due to its time-modeling abilities. Applying the above modifications may further improve diagnostic and communication methods for people suffering from ABI employing future generation computers.

## Methods

### Research design

The experiment consisted of 2 stages. First, a hearing test based upon objective ABR method was conducted to find out, if participants will be able to understand spoken commands from a therapist during a therapeutic session. Next, a series of mental exercises conducted by a therapist took place. During the exercise, a set of activities such as looking at the word spoken by a therapist and simultaneously displayed on the computer monitor was performed. EEG and EGT signals were recorded during each exercise. The result of each session was an anonymized data set consisting of patient data, an EEG recording, an EGT recording, and the answers given to questions asked by the therapist.

The second stage of the experiment helped to extract a set of parameters obtained from the EEG and EGT signals performed in order to find a correlation of these values with the Glasgow Coma Scale (GCS) scores of participants.

### Participants

All experiments presented in this paper were carried out in the “EPIMIGREN” Neurorehabilitation Medical Centre in Osielsko, Poland. The data were collected during three periods. A total of 33 participants were involved in the study: 10 participants in the first series of experiments, 13 in the second series, and 10 participants in the third one. Experiments were conducted in periods between 12.12.2016–31.01.2017 (A), 2017/05/29–2017/07/13 (B), and 2018/01/22–2018/03/15 (C). The average age of the participants was 45.5 years. The state of the participants’ consciousness was assessed using the GCS subjective evaluation scale. GCS is one of the most popular methods of subjective assessing the consciousness of people with brain injuries, consisting of an evaluation of eye-opening, the best verbal answer and the best motor reaction caused by external stimuli [23, 24]. The participants are awarded several points, from 3 pt (related to deep unconsciousness) to 15 pt (reflecting a mild dysfunction). Both the components of the score and their sum were used for further analysis. Table 3 presents the demographic data of the participants and their GCS scores.

**Table 3:** Basic information about participants and their GCS assessment results

Subject ID	Age range	Cause	Interval post-ictus (months)	Best eye response	Best motor response	Best verbal response	GCS
01	51–60	Sudden cardiac arrest	17	2	3	1	6
02	41–50	Traffic accident	21	3	3	1	7
03	21–30	Sudden cardiac arrest	17	3	2	2	7
04	21–30	Traffic accident	24	4	3	1	8
05	51–60	Fall down the stairs	26	2	3	3	8
06	41–50	Sudden cardiac arrest	10	4	3	2	9
07	51–60	Sudden cardiac arrest	12	4	4	1	9
08	21–30	Traffic accident	37	4	4	2	10
09	51–60	Sudden cardiac arrest	22	4	4	2	10
10	41–50	Fall from a ladder	9	4	4	3	11
11	61–70	Sudden cardiac arrest	10	2	2	1	5
12	41–50	Stroke	1	2	2	1	5
13	51–60	Fall down the stairs	18	2	3	1	6
14	41–50	Traffic accident	44	2	3	2	7
15	51–60	Sudden cardiac arrest	22	2	3	2	7
16	21–30	Sudden cardiac arrest	22	3	2	2	7
17	21–30	Traffic accident	31	4	2	1	7
18	31–40	Sudden cardiac arrest	5	4	1	2	7
19	41–50	Traffic accident	26	3	3	2	8
20	61–70	Stroke	7	3	3	2	8
21	61–70	Traffic accident	13	3	3	1	7
22	21–30	Traffic accident	43	4	4	2	10
23	51–60	Stroke	27	4	4	2	10
24	51–60	Sudden cardiac arrest	29	2	2	1	5
25	61–70	Stroke	14	4	3	1	8
26	31–40	Sudden cardiac arrest	12	4	2	1	7
27	51–60	Sudden cardiac arrest	24	4	1	1	6
28	51–60	Sudden cardiac arrest	35	4	4	2	10
29	21–30	Sudden cardiac arrest	29	3	3	2	8
30	41–50	Traffic accident	33	4	3	1	8
31	61–70	Sudden cardiac arrest	8	4	3	1	8
32	11–20	Sudden cardiac arrest	51	3	3	1	7
33	31–40	Traffic accident	31	4	2	1	7

### Measures

Ground truth data related to the state of consciousness of participants were acquired from the expert therapist in the form of GCS scores (in terms of both the partial scores and their sums). The Pearson correlation coefficient was employed to compare GCS and parameters obtained from the unsupervised machine-learning, namely, to the analysis of EEG and EGT signals as it is presented in detail in a subsequent part of the paper.

### Procedure

We introduced a multi-stage method in our study for multimodal monitoring of ABI participants’ performance employing human–computer interaction. First, the hearing abilities of the participants were tested using auditory brainstem response measurements. This stage allowed for the selection of participants whose hearing abilities were good enough to enable them to understand the verbal commands of the therapist during the mental exercise session. The detailed results obtained for 23 people in the course of the experiments are presented in our paper. Also, 32 participants took part in exercise sessions with the therapist employing a multimedia computer equipped with a gaze tracker and an EEG helmet. The exercises were based upon simple tasks performed using an eye-tracker controller. The participants were encouraged to look at the image displayed on the computer monitor specified by the therapist or to fill a gap in a sentence with one of three words provided. The brain activity of participants was monitored in the course of each session. The data gathered from the eye tracker and the EEG headset were analyzed using a multimodal autoencoder neural network, which is an unsupervised machine learning algorithm use, i.e. for the task of assessing separability of classes in the datasets or learning parameterization techniques which are tailored to a particular problem. We decided to employ a so-called multimodal autoencoder which in addition to the parameterization and clustering of data in the decision space is capable of learning the method to perform a fusion of information gathered from multiple modalities which in case of our study are signals from EEG and EGT [25, 26]. There are many recent examples of the use of autoencoders for such a purpose, i.e. in the field of robotics [27–29].

An advantage of multimodal autoencoders is that they can produce a vector of parameters based on the fusion of data originating from two or more different modalities. The way this fusion is performed is found and optimized during the training of the autoencoder.

Unsupervised machine learning and data exploration algorithms were already used to evaluate the emotional states of participants [30–32], as a diagnostic decision support mechanism in the process of epilepsy treatment [33], to provide a way of controlling various devices by disabled people [34], and as a basis for stroke rehabilitation [35]. Estimation of brain states and activities based on data obtained from biosignal monitoring devices is a challenging concept if people with communication disorders are considered. The estimation of mental activities of the participants together with information related to their performance in the mental exercise sessions may provide a valuable source of information for the therapist deciding on further treatment. Similar mental activities may be defined for instance, in terms of a content of a particular set of values derived from EEG headset used for the monitoring of participant activity. Such an approach may address the problem of evaluating mental activities or mental abilities of people with neurological disorders or those after brain injuries. The monitoring of mental activity can even enhance communication with them by using brain–computer interface (BCI) devices [36]. Also, the interaction of such people with a computer often requires the usage of some form of BCI such as an eye tracker. Such an interface also becomes a good tool for monitoring the state of such person by analysis of the way it is used for communication with the computer. It may also provide a potential method to compare the GCS score of a patient with the outcomes of assessment made on the base of his or her performance during computer-based mental exercises. In turn, such a measure may be used as a means for tracking patients’ progress, which could be helpful for a therapist, especially if GCS is not used or was replaced by other measures [37].

### Hearing tests

**Fig. 6:**
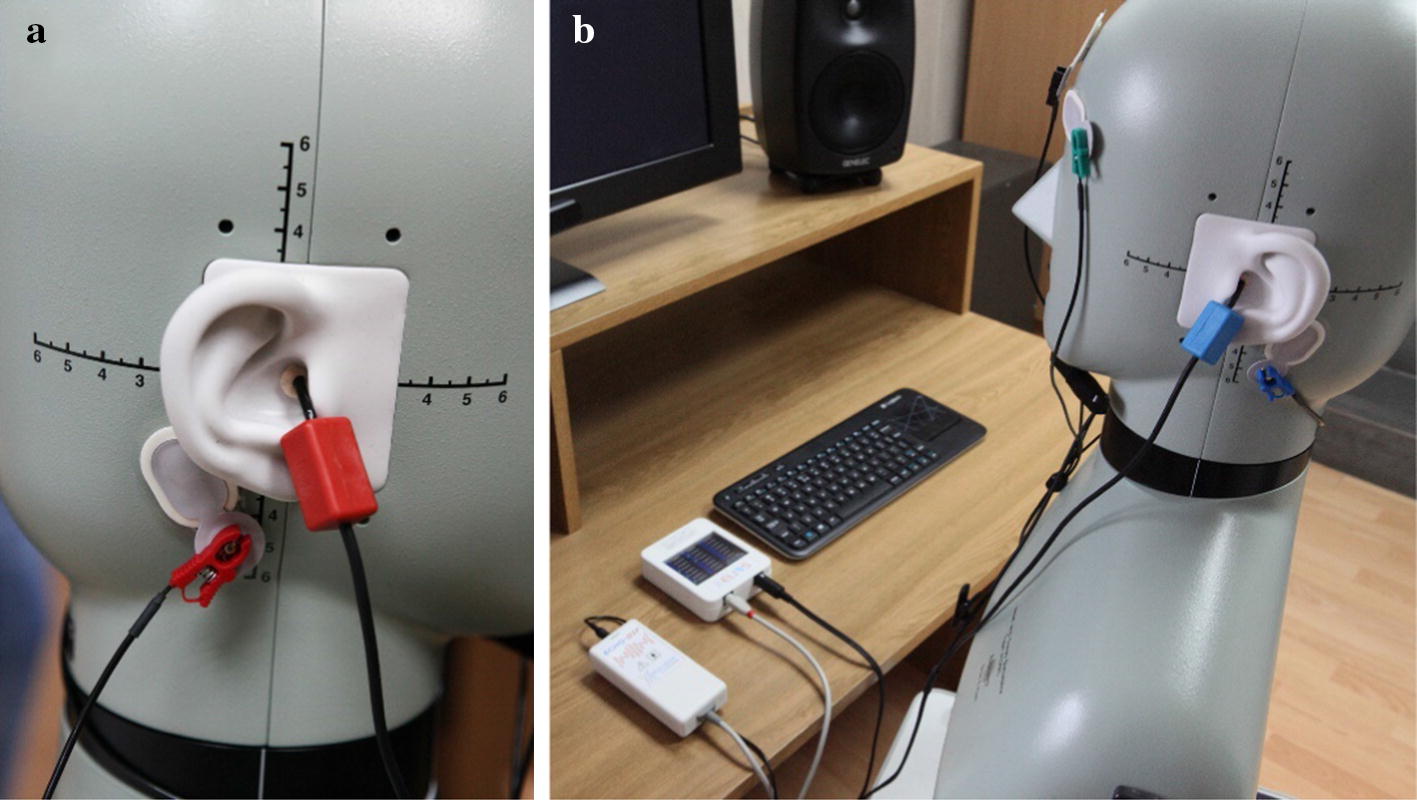
Device for ABR measurement showing **a** the electrode mounted on the right mastoid and in-the-ear headset; **b** the left-side electrodes and device units (visible on desktop) [40]. The data collected can easily be imported to a personal computer for analysis

The first step in the evaluation of a participant with ABI was to verify hearing ability. The ability to hear, and therefore to receive and understand commands, suggestions, and guidelines, is a prerequisite for stimulation using spoken commands. In the present study, auditory evoked potentials (ABR—auditory brainstem response) were chosen for assessing hearing abilities of participants, since this method of assessment has numerous advantages: it is non-invasive, painless, and does not involve any complex preparation [38, 39]. The Echodia Elios device was used for ABR measurements, which is portable (Fig. 6), its parameters can be set with a touchscreen, and the results are saved in the built-in database [40].

The measurement parameters were as follows:

- stimulus: a click;
- number of stimuli (for each level and each ear): 1000;
- number of clicks per second: 17;
- stimulus levels (first series): 60, 40, 25, 10 dB nHL (nHL—normal adult hearing level);
- stimulus levels (second series): 90, 80, 70, 60, 50, 40, 30 dB nHL.

In the second series, the stimulus level range was broadened in order to assess the participants’ hearing ability more accurately. A louder stimulus induces stronger responses, which should facilitate the process of the fifth wave detection. The 10-dB nHL level was skipped since the values of the recorded responses were a very low and subjective analysis of the plot might, therefore, have suffered due to severe inaccuracies and, as a consequence, responses obtained for this stimulus during the first series of the study were also omitted.

The device automatically rejected answers to stimuli affected by artifacts coming from participants’ muscular activity (in that case, the stimulus was automatically repeated). Therefore, the time of any particular measurement varied between 10 and 18 min, depending on the number of rejected answers detected by the device.

**Fig. 7:**
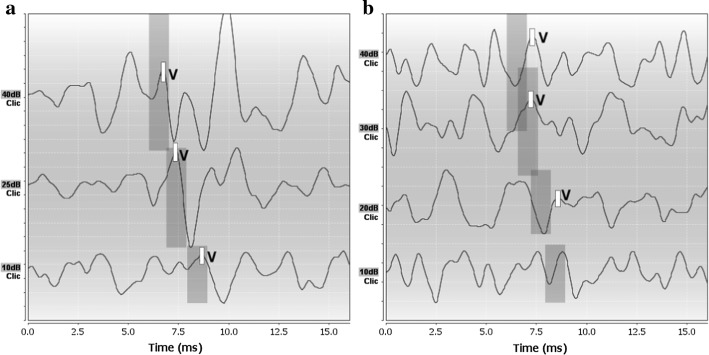
ABR results in a comparison of **a** healthy person and **b** participant 07. Grey rectangles indicate reference latencies of the 5th wave

The analysis of recorded ABR can be used to evaluate the participants’ auditory pathway. This feature of the ABR was especially important for the current research. The ABR morphology of all the participants revealed malfunctions in the auditory pathway. The shape of the responses, amplitudes, and latencies of waves significantly differed from those observed in healthy participants. Figure 7 illustrates the differences between responses observed in a healthy participant and a participant with ABI (participant 07).

It was particularly difficult to determine the exact position of the fifth wave in the brainstem response received, thus the final results may suffer from some inaccuracies caused by the subjective analysis of the plot. This issue applies particularly to the lowest stimulus intensity (10 dB nHL) since the values of responses recorded were very low in that case. Nevertheless, some important observations can be made based on the results obtained. The latencies of the 5th wave (Tables 4 and 5) were significantly longer than those observed in healthy participants.

**Table 4:** Latencies of the 5th wave for subjects 01–10

Subject ID	Latency (ms)—right ear			Latency (ms)—left ear		
c.i.	60	40	25	60	40	25
01	5.25	6.63	7.09	5.34	6.75	8.28
02	6.72	8.06	8.44	6.41	6.97	8.03
03	5.31	7.56	8.38	5.50	7.63	8.00
04	5.88	7.34	8.56	5.81	7.59	8.91
05	6.72	8.66	8.88	–	8.88	9.72
06	6.16	6.34	7.72	5.63	6.63	7.19
07	7.34	7.28	8.59	6.41	7.31	9.19
08	6.22	6.59	8.69	7.25	7.91	9.66
09	6.03	7.31	8.19	6.09	7.19	8.09
10	6.63	8.00	9.78	6.66	8.03	9.78

c.i. denotes click intensity (dB HL)

**Table 5:** Latencies of the 5th wave for subjects 11–23, c.i. denotes click intensity [dB HL]

Subject ID					Latency (ms)—right ear					Latency (ms)—left ear				
c.i.	90	80	70	60	50	40	30	90	80	70	60	50	40	30
11	5.34	5.56	5.78	6.44	6.44	6.56	7.28	4.91	5.5	5.88	6.31	6.81	–	–
12	6,00	6.25	6.63	7.38	7.59	8.28	8.19	–	7.09	6.34	–	–	–	–
13	5.06	4.88	5.03	5.16	6.34	7.22	8.09	5.41	5.28	6.38	6.19	7.28	–	–
14	6.63	6.72	6.81	6.81	7.06	7.06	7.22	5.16	5.47	5.66	5.91	6.16	7.19	7.44
15	4.81	5.66	5.47	5.75	6.25	6.94	7.16	5.41	6.06	5.97	6.44	7.19	7.75	8.22
16	5.69	5.59	6.28	6.41	6.91	8.5	–	6.16	6.22	6.66	6.72	–	–	–
17	5.22	5.59	5.69	5.47	6.22	7.41	–	5.06	5.47	6.53	6.97	–	–	–
18	5.00	5.72	6.13	–	6.88	–	7.53	4.91	5.28	5.5	6.13	6.81	7.09	–
19	5.63	5.81	6.16	6.31	7.56	7.75	–	5.69	5.56	5.81	–	–	6.91	–
20	5.84	5.47	6.19	6.66	7.28	7.66	9.09	5.34	6,00	6.19	6.59	6.75	7.94	–
21	5.81	6.13	6.22	6.94	7.91	8.16	–	6.28	6.84	7.34	8.28	8.72	9.69	–
22	5.09	5.53	6.00	6.03	6.28	6.72	7.25	5.59	5.53	5.97	6.38	6.41	6.56	6.75
23	5.88	5.84	6.03	6.59	7.09	7.16	7.59	5.56	5.69	6.09	6.41	6.72	7.22	8.03

**Fig. 8:**
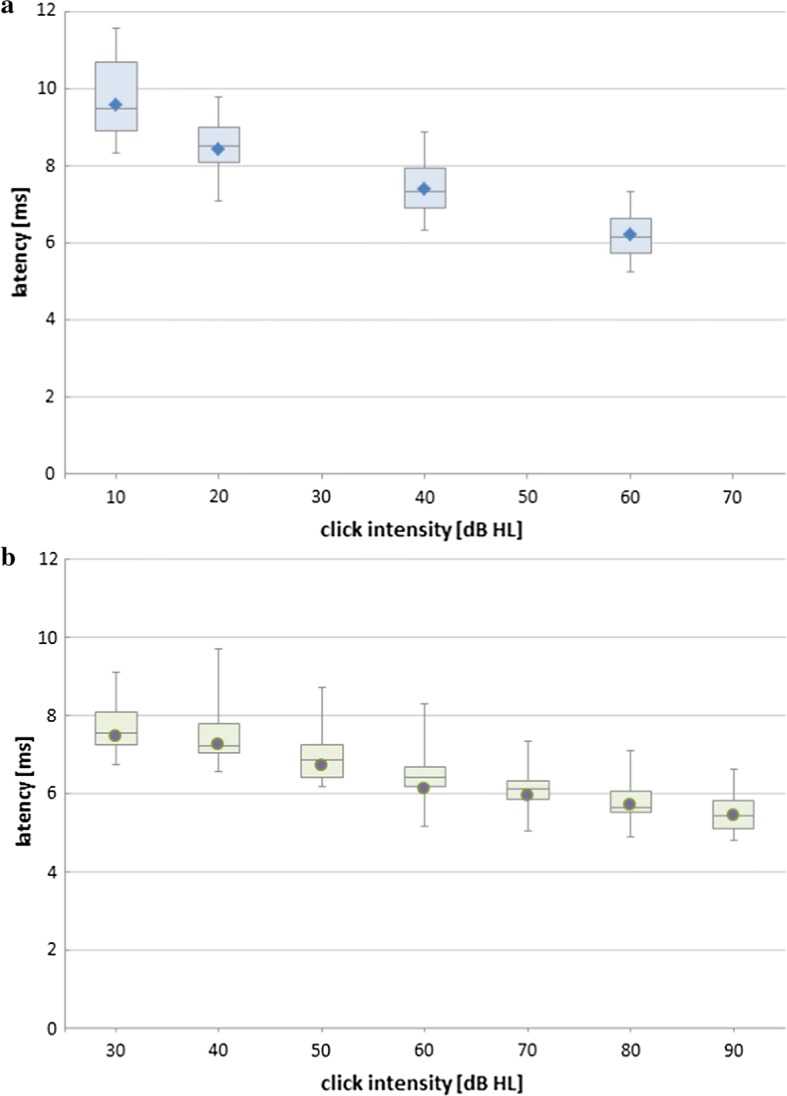
Latencies of the 5th wave from all participants plotted against stimulus intensity, **a** participants 1–10, **b** participants 11–23

However, with decreasing stimulus intensity, latencies elongated systematically (Fig. 8). The above effect was also observed in healthy participants. To conclude, the results of the participants with ABI showed significant abnormalities in the auditory pathway, but they did not seem to exclude the possibility of verbal communication with the participants.

### Assessment of participants’ performance through classification of mental activities

**Fig. 9:**
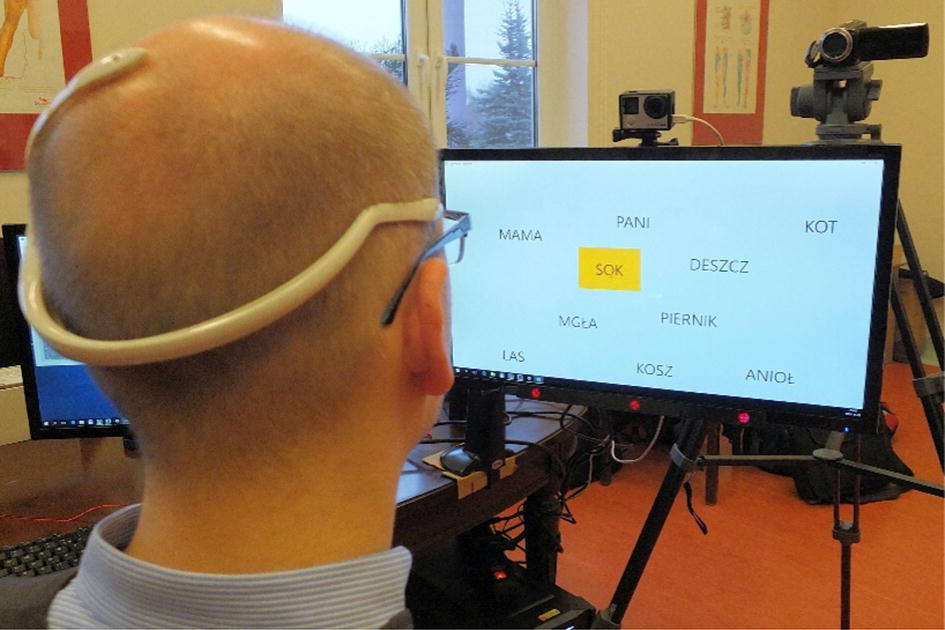
The multimodal stand used for performing mental exercises and experiments with participants suffering from traumatic brain injuries. The system programmer tests its operational readiness

To discover if participants were able to be mentally involved in activities initiated by the therapist, a set of activities was conceived. They are associated either to solving simple puzzles like filling a gap in a sentence with one of three possible words shown on the computer screen or to answering questions asked by the therapist by gazing at the correct answer on the screen, i.e., “yes” or “no” or through selecting displayed objects. The fixation of eye-gaze lasting for longer than 2 s was interpreted as an act of choosing the indicated option. All the activities were performed using the eye-tracker device by Tobii EyeX [41]. According to the eye-tracker device technical specification, fixation point coordinates are recorded with more than 60 samples/s. The device was mounted at the bottom of the computer monitor and then used for collecting information related to the areas of the screen that drew the attention of the participant. The participant was located at about 60 cm distance (head to screen surface). The multimodal system for experimenting is depicted in Fig. 9.

The setup consists of an EEG headset (placed on the head of the participant), eye tracker, monitor, and video cameras for capturing the overall course of the experiment. The system was controlled by a computer equipped with the developed software for providing stimuli and performing data acquisition (the words displayed are in Polish).

Emotiv INSIGHT and Emotiv EPOC EEG headsets [42–44] were used for acquiring data related to the brain activity of participants while they performed specified tasks. The INSIGHT headset [43] consisted of five signal electrodes, AF3, AF4, P7, P8, Pz, and a single reference electrode; the sensors were dry-type. The EPOC EEG headset allowed us to gather data from 14 electrodes, AF3, AF4, F7, F3, FC5, T7, P7, O1, O2, P8, T8, FC6, F4, F8 (following standardized electrode marks). The above device was equipped with saline-based electrodes. Data were transmitted via a wireless connection to a PC equipped with a USB receiver dongle. The sampling frequency of output signals was equal to 128 Hz. The software provided by the headset manufacturer was employed to get samples of each electrode signal, whereas a self-developed software stored them to .csv file for the usage at further stages of the analysis.

An automatic system for providing various types of tasks and for collecting feedback from the therapist was prepared. A detailed description of each activity, together with its identification number, is listed in Table 6. The ID numbers of activities were employed for the analysis in subsequent parts of this paper to indicate a set of activities performed by a participant in each session described.

**Table 6:** ID numbers and types of activities performed by subjects with the use of eye tracker

Activity ID	Description of the activity
1	Typing on the virtual keyboard
2	Putting down letters in words associated with displayed images
3	Rewriting a word presented on the screen
4	Selecting a word spoken by a therapist
5	Selecting of a sentence spoken by a therapist
6	Matching missing words to gaps in the sentences
7	Selecting images specified by a therapist
8	Answering yes/no through displayed panel

The activity of the participant was monitored using an eye-tracker device and cameras. The therapist who had control throughout the session could also report various events that occurred during each performed activity by clicking on appropriate buttons displayed on the control screen of the application. Events were automatically logged in the data file produced by the eye tracker and linked to the record of the current position of the fixation point. Such events could be tracked later on in the post-processing stage and visualized as annotations on the processed EEG signals. The eye tracker has to be calibrated before the experiment. For majority participants with ABI, it is very hard or even impossible to perform calibration of the device through the process consists of fixating the eye gaze on selected points in the corners of the screen lasting about 4 s. In turn, the necessity to follow calibration points by gaze often makes it impossible for participants to perform the calibration. Therefore, the therapist had to plan where the participant will be positioned during the experiment in order to calibrate the device while occupying the same place as the person engaged in the experiment. Events that could be reported in this way were: beginning and ending of an identified exercise, somebody entering the room during the session, the pain felt by the participant, or movement of the participant, among others.

A set of results gathered from 85 therapeutic sessions was collected. Data from electrodes of the headset were stored in the .csv and .edf file formats. The data consisted of EEG records associated with the brain activity of participants, data from the eye-tracker device, and logs of events reported by the therapist. Both 5-channel and 14-channel data were taken into consideration during the post-processing and machine learning-based analysis stage.

**Fig. 10:**
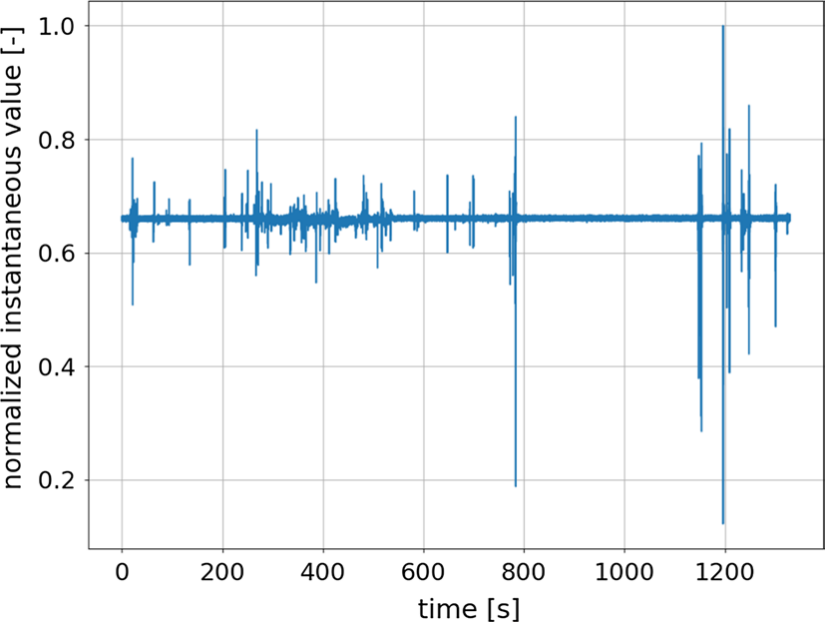
Example of the signal acquired from a single electrode (AF3) of the 5-electrode EEG headset with electrodes having good contact with the subject’s skin

**Fig. 11:**
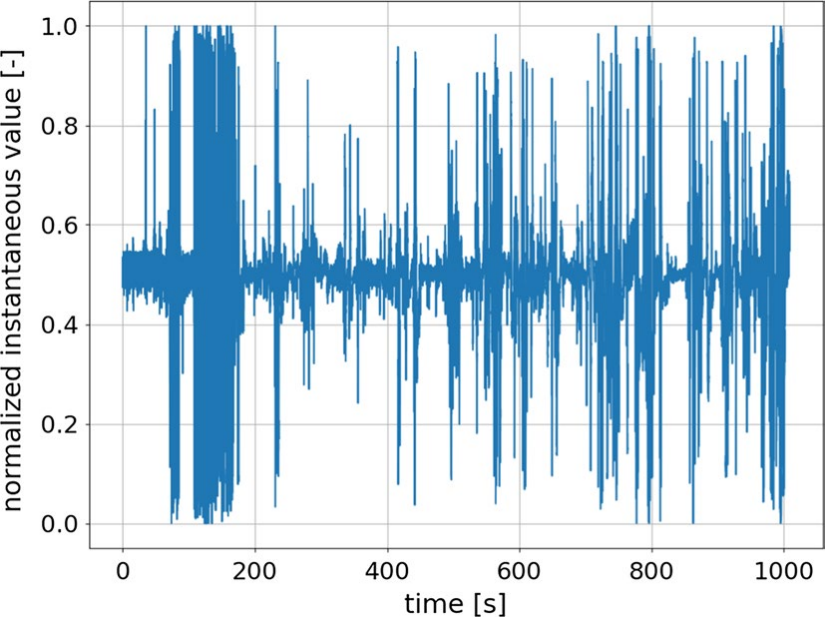
Example of signal gathered from a single electrode (AF3) of the 5-electrode EEG headset with electrodes having bad contact with the subject’s skin

Such an approach to signal acquisition permitted to analyze data obtained from both headsets with a single algorithm. One of the problems which were spotted in the initial stage of research is differences in the quality of signals obtained from individual participants. The quality varied depending on the shape of the head of the participant taking part in the experiment and on skin conductivity. Problems with skin contact were especially prominent in the 5-electrode headset. The main problem was noise occurrence caused by the lack of perfect contact between electrode and participants’ skin. Example of a signal containing only spike-like artifacts occurring due to a sudden loss of contact between the skin an the electrode is depicted in Fig. 10. An example of a signal degraded by noise is shown in Fig. 11.

We decided to leave also the signals containing visible artifacts in the dataset, as the next step of the processing would involve the use of unsupervised machine learning algorithms—a multimodal autoencoder. One of the features of such an algorithm is its ability to find a general model for even a noisy data output. Our dataset also consisted of a large number of epochs which do not contain any artifacts (especially ones utilizing 14 saline-based electrodes). Also, presence of noise is one more reason to process the data with the autoencoder algorithm, as its bottleneck-type structure forces it to seek the best, simple model of data that is received during the training. Noise reduction is one of common applications of autoencoders [45, 46].

### Post-processing of EEG signals

Computations were performed using software written in Python programming language extended by several scientific calculation libraries, including SciPy [47], NumPy [48], Keras [49], and TensorFlow [50]. The input signal obtained from the EEG headset consisted of 5 or 14 electrodes, depending on the type of headset used for the data acquisition. The common electrodes between both headsets are AF3, AF4, P7, and P8. Therefore, to unify the format of all signal sets—in place of all signals which were not gathered by a headset, a placeholder signal consisting of a sequence of zeros was introduced. After this step, each set of EEG signals consisted of 15 channels, and it contained either 10 or 1 placeholder signals.

**Fig. 12:**
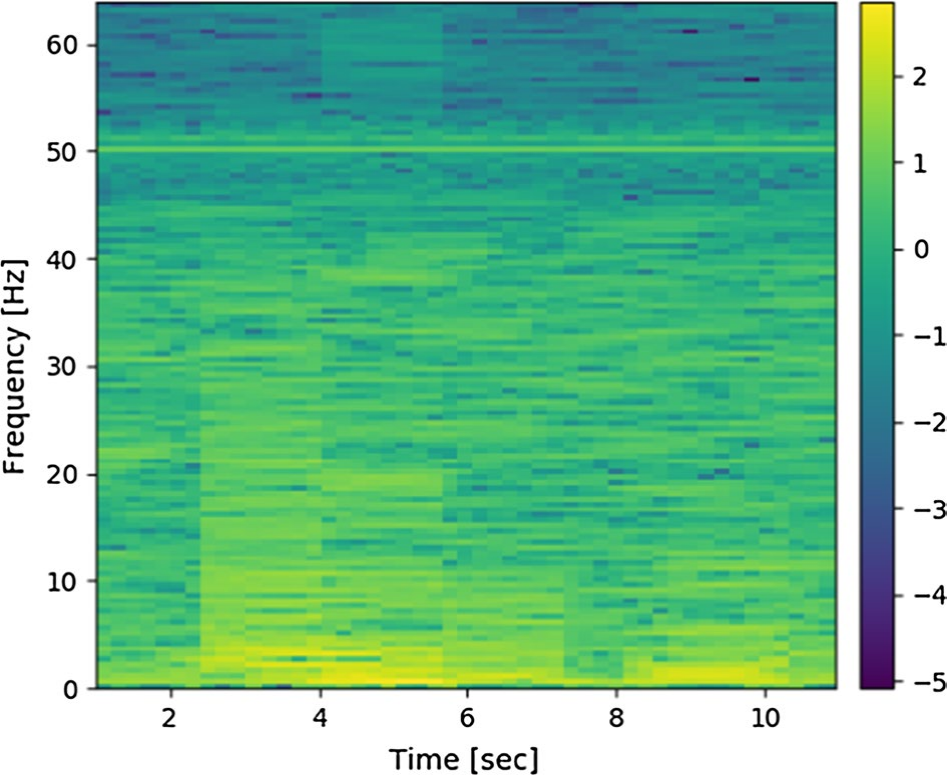
Example of spectrogram calculated for the epoch obtained from the T8 electrode of the 5-electrode headset

The next step was to split EEG signals into epochs. No overlap was employed for this part of processing. Information about the time of beginning and ending of each frame was also kept to allow synchronization of EEG signal epochs with analogous epochs of signals gathered from the eye tracker. The whole dataset after pre-processing stage consisted of 9436 epochs of EEG and eye-tracker signals. Each of them lasted 12 s, therefore all the epochs are equivalent to 31.5 h of continuous data acquisition. After splitting the signals into epochs, for each channel of EEG in each epoch, a spectrogram was calculated. The size of FFT employed for this task was 256, the overlapping factor of 0.9 was employed. As a windowing function, a Tukey window with a shape parameter of 0.25 was used. It is a default window used in the SciPy library procedure employed for the task of this calculation. Each spectrogram was standardized according to the formula (2):

2 $$S\left( {f,t} \right) = \frac{{S\left( {f,t} \right) - \overline{{S\left( {f,t} \right)}} }}{{{\text{std}}\left( {S\left( {f,t} \right)} \right)}},$$

where $S\left( {f,t} \right)$ denotes a spectrogram which is a function frequency $f$ and time $t$. The mean value of the spectrogram averaged over time and frequency is denoted by $S\left( {f,t} \right)$, and standard deviation of the spectrogram is denoted by ${\text{std}}\left( {S\left( {f,t} \right)} \right)$. An example of spectrogram calculated for one of EEG epochs is depicted in Fig. 12.

### Post-processing of eye-tracker data

**Fig. 13:**
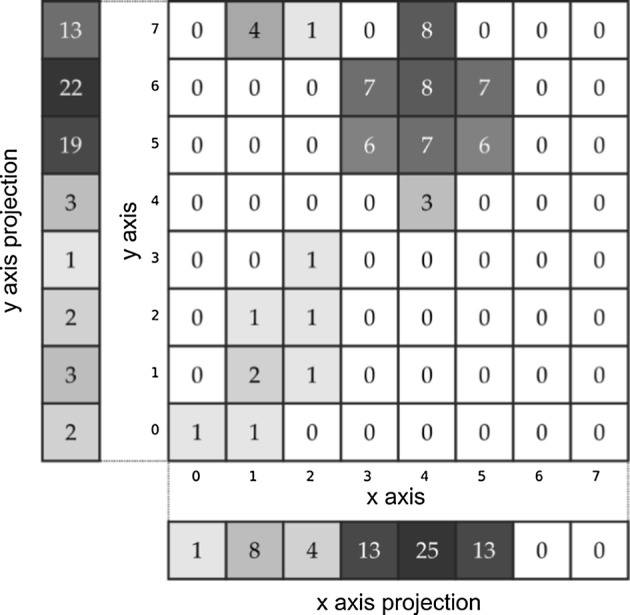
Example of calculation of a heatmap for a given matrix of values

Another tool used for monitoring the behavior of the participants was the eye tracker. Results obtained from this device are a series of data points containing 3 coordinates, namely: x and y coordinates of eye-fixation point at the given moment, plus the time index. Eye-tracker signal was initially split into epochs in such a manner, that it always corresponded to one of the frames of EEG signals. Each of these epochs was then further processed by the post-processing algorithm. Data contained by each of eye-tracker signal epochs may be visualized by plotting the trajectory of eye-fixation point movement. However, the form of visualization is illegible if long trajectories are plotted. Therefore, the data were processed in such a way that for each pixel of the computer screen, a frequency of eyepoint fixation occurrence at this point was calculated. The matrix of such values may be treated as an estimate of the probability that the participant was looking at a certain pixel. The matrix may be displayed in a graphical form. This type of visualization is called heatmap. To derive parameters from the 2D graphical representation of the eye-gaze position, projections of the heatmap on the *x*-axis and *y*-axis of the coordinate system were calculated. Illustration of the process and the procedure of displaying the heatmap is shown in Fig. 13.

**Fig. 14:**
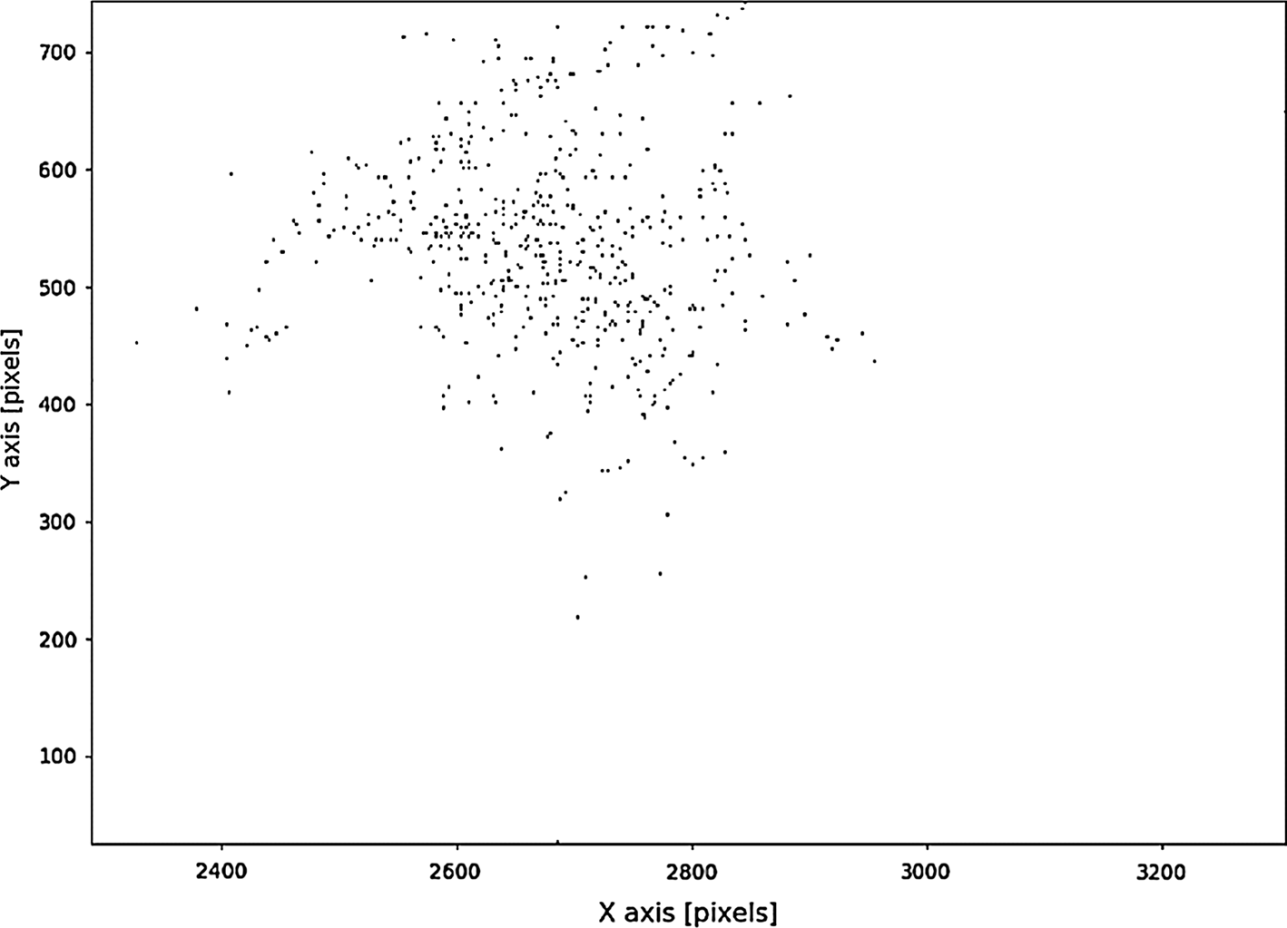
Example of spectrogram calculated for the epoch obtained from the T8 electrode of the 5-electrode headset

As eye-tracker signal in such a form is represented simply by a two-dimensional matrix, it is a convenient form of data to be used as an input for the convolutional neural network, which consisted of an autoencoder network employed in our study. Example of heatmap calculated from the data obtained during the experiment is depicted in Fig. 14.

### Analysis of EEG and eye-tracker signals with the multimodal autoencoder neural network

**Fig. 15:**
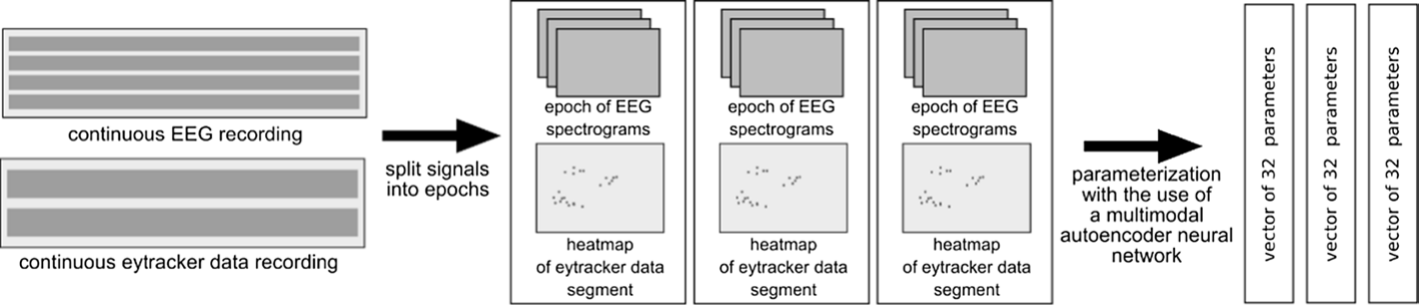
Schematic depiction of EGT and EGT signals post-processing and formation of data frames analyzed by the autoencoder neural network

The algorithm of the multimodal autoencoder used to analyze the data from the experiment took as an input the pairs of signal epochs obtained from the EEG and EGT acquisition devices. Each pair of post-processed epochs is processed by the neural network, and the effect of this processing is a vector of 32 floating-point numbers which can be treated as a vector of parameters assigned to each pair of the epoch by the neural network. The particular way of assignment of those vectors is found up by the autoencoder algorithm itself during its training. Schematically, the process of the postprocessing of those signals may be depicted as in Fig. 15.

Methods of data analysis with embeddings calculated with use of neural networks or other techniques of were proven to be useful in many applications such as text or image processing [51, 52]. There are also examples of such techniques applied to medical data [53, 54]. Auto-extracted features of medical signals were processed with use of methods such as PCA, which may be applied as a tool for visualization of high-dimensional feature vectors calculated by neural networks. The more detailed analysis may be performed with use of standard statistical methods such statistic tests. The approach based upon statistics is more precise in this case because it can be performed without dimensionality reduction, which is caused by use of PCA. In our work we use PCA only for the purpose of visualization. Calculation associated with statistical analysis is performed on data derived from high-dimensional feature vectors (embeddings) generated by employed neural networks.

In the case of our experiment, data from neural network inputs are processed by consecutive layers of the network. After certain amounts of layers, intermediate results of calculations made for EEG and EGT signals are merged and from that point processed together to produce a single vector of parameters consisting of 32 floating-point numbers.

**Fig. 16:**
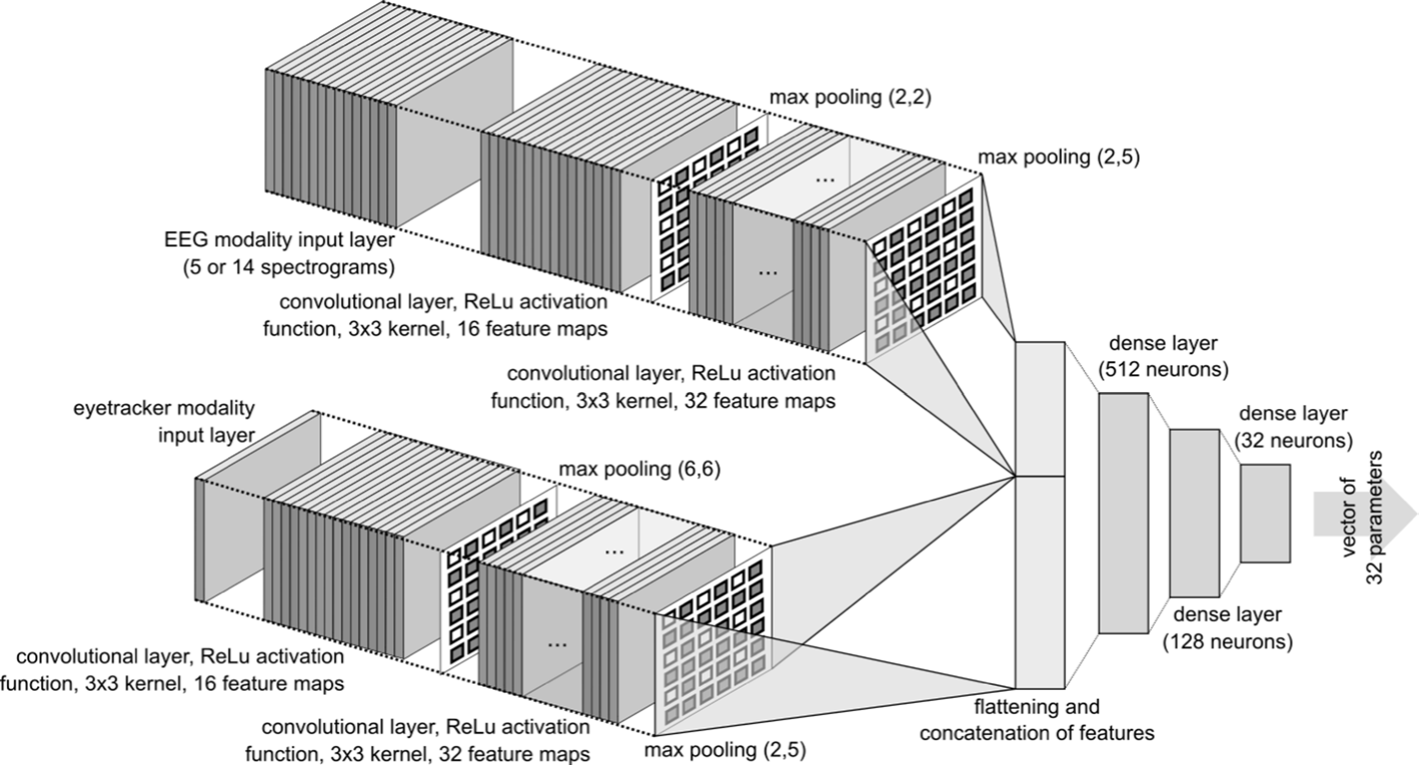
Example of spectrogram calculated for the epoch obtained from the T8 electrode of the 5-electrode headset

**Fig. 17:**
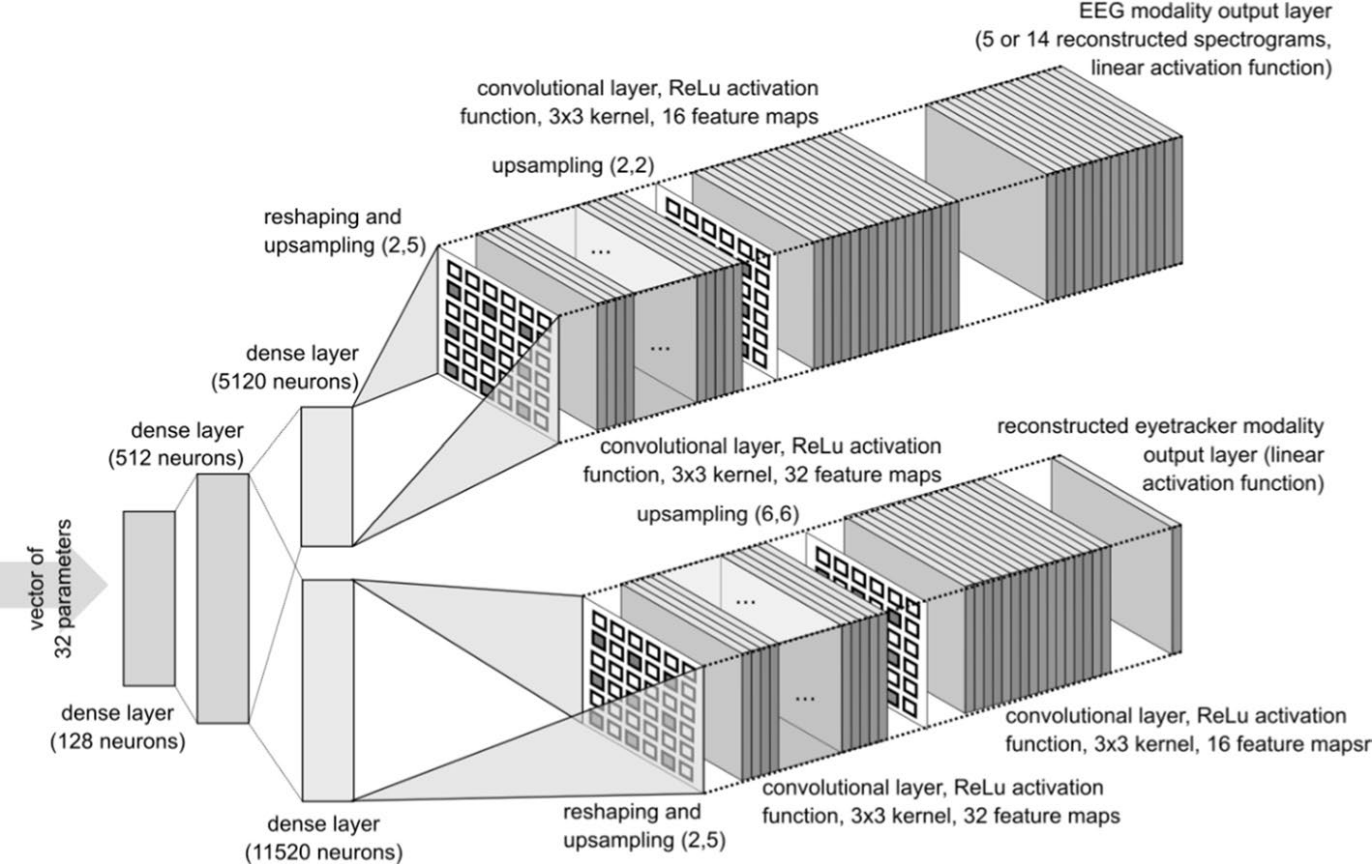
Example of spectrogram calculated for the epoch obtained from the T8 electrode of the 5-electrode headset

The decoder part of the autoencoder usually has an inversed structure when compared to the encoder, as it performs the inverse operation on the vector of parameters generated by the encoder. The goal during the training of the autoencoder is to minimize the error measured between the input of the encoder and the reconstructed versions of the input obtained from the decoder. In such a way, the autoencoder learns to encode information about the input on the limited number of parameters of which the result vector of parameters consists. The architecture of the encoder and decoder parts of the neural network employed in our study is depicted in Figs. 16 and 17. They also provide details of layers used, their activation functions, and numbers of feature maps in each layer of the convolutional subnetworks.

The EEG signal representation is provided to the network as a 5-channel or 14-channel input layer of a convolutional neural network. The exact number of channels depends on whether the input dataset is one obtained with the use of 5-channel or 14-channel EEG headset. The EGT input is a single-channel input layer of the convolutional network. The whole dataset used for training and further analysis of vectors of parameters generated by the autoencoder consisted of 5006 pairs of EEG and EGT signal epochs in case of dataset related to the 5-electrode headset and 3000 pairs in case of one related to the 14-electrode headset.

In each case, the training set consisted of 90% of examples and validation set consisted of 10% of all examples. The advantage of employing the autoencoder algorithm is the fact that it can process heterogeneous data as an input. This is a kind of data we have in our study because they were acquired with the use of two different headsets. The autoencoder is also capable of learning the way of fusion of information obtained from the EEG and EGT modalities and encode it in a single vector of parameters. Stochastic gradient descent was used as a training algorithm, the ADAM learning rate optimization method was employed. The base learning rate for the ADAM optimizer was set to $10^{ - 4}$, the rest of the parameters were set to their defaults in the Keras library implementation. The mean squared error was used as a loss function for the optimization algorithm. The total value of the loss was the weighted sum of losses calculated for the two separate outputs of the autoencoder. The weight of the loss of a loss component related to EEG output was equal to 1, the weight of loss component related to EGT was set to 100. The batch size was equal to 128. The algorithm was trained for 200 iterations, each of them lasted for 9 s. Therefore, the total duration of the training was approximately equal to 1 h. The training was performed on Titan RTX graphics card.

## Data Availability

The datasets used and/or analyzed during the current study are available from the corresponding author on reasonable request.

## Abbreviations

EEG: electroencephalography
ABR: auditory brainstem response
EGT: eye-gaze tracking
GCS: Glasgow Coma Scale
TBI: traumatic brain injury
SCA: sudden cardiac arrest
ABI: acquired brain injury
CT: computed tomography
MRI: magnetic resonance imaging
PET: positron emission tomography
BCI: brain–computer interface
nHL: normal adult hearing level
ICA: independent component analysis
DWT: discrete wavelet transform
